# 2D Bi_2_Se_3_ van der Waals Epitaxy on Mica for Optoelectronics Applications

**DOI:** 10.3390/nano10091653

**Published:** 2020-08-22

**Authors:** Shifeng Wang, Yong Li, Annie Ng, Qing Hu, Qianyu Zhou, Xin Li, Hao Liu

**Affiliations:** 1 Department of Physics, Innovation Laboratory of Materials for Energy and Environment Technologies, College of Science, Tibet University, Lhasa 850000, China; xzuliyong@utibet.edu.cn (Y.L.); zhouqianyu@utibet.edu.cn (Q.Z.); lixin@utibet.edu.cn (X.L.); liuhao@utibet.edu.cn (H.L.); 2 Institute of Oxygen Supply, Center of Tibetan Studies (Everest Research Institute), Tibet University, Lhasa 850000, China; 3 Department of Electrical and Computer Engineering, Nazarbayev University, Nur-Sultan 010000, Kazakhstan; 4 School of Environmental Science and Engineering, Southern University of Science and Technology, Shenzhen 518055, China; huq@sustech.edu.cn; 5 Engineering Innovation Center of Southern University of Science and Technology, Beijing 100083, China

**Keywords:** van der Waals epitaxy, Bi_2_Se_3_, mica, two-dimensional materials, optoelectronics, transparent conductive electrode

## Abstract

Bi_2_Se_3_ possesses a two-dimensional layered rhombohedral crystal structure, where the quintuple layers (QLs) are covalently bonded within the layers but weakly held together by van der Waals forces between the adjacent QLs. It is also pointed out that Bi_2_Se_3_ is a topological insulator, making it a promising candidate for a wide range of electronic and optoelectronic applications. In this study, we investigate the growth of high-quality Bi_2_Se_3_ thin films on mica by the molecular beam epitaxy technique. The films exhibited a layered structure and highly c-axis-preferred growth orientation with an XRD rocking curve full-width at half-maximum (FWHM) of 0.088°, clearly demonstrating excellent crystallinity for the Bi_2_Se_3_ deposited on the mica substrate. The growth mechanism was studied by using an interface model associated with the coincidence site lattice unit (CSLU) developed for van der Waals epitaxies. This high (001) texture favors electron transport in the material. Hall measurements revealed a mobility of 726 cm^2^/(Vs) at room temperature and up to 1469 cm^2^/(Vs) at 12 K. The results illustrate excellent electron mobility arising from the superior crystallinity of the films with significant implications for applications in conducting electrodes in optoelectronic devices on flexible substrates.

## 1. Introduction

Since the discovery of graphene by Geim and Novoselov in 2004 [1], two-dimensional materials have gained renewed interest, because of their exceptional electronic properties, low dangling bond density, and high specific surface areas that are important for optoelectronics, sensing, catalysis, and energy storage applications. Recently, transition metal dichalcogenides (TMDs), such as MoS_2_, WoS_2_, and Bi_2_Se_3_, have become the research focus. The latter material is of particular interest as a topological insulator and thermoelectric material [2,3,4,5,6]. Layered Bi_2_Se_3_ possesses an insulating bulk gap of 0.3–0.35 eV and metallic surface states with a single Dirac cone [7,8,9,10], enabling excellent transport properties with a high carrier mobility [11]. In addition, the surface conduction can be significantly boosted and easily tailored in few-layer nanostructures consisting of large surface-to-volume ratios [12,13,14,15]. Being analogous to the optoelectronic applications of graphene, a thin layer of Bi_2_Se_3_ has been proved to be a promising material for broadband and high-performance optoelectronic devices, such as photodetectors, terahertz lasers, and transparent electrodes [3,16]. In particular, transparent conductive electrodes (TCE) play a vitally important role in modern optoelectronic devices, including displays, light-emitting diodes, touch screens, and photovoltaics. Doped metal oxides, such as indium tin oxides (ITO), are predominantly utilized for such applications, owing to their high electrical conductivity and good optical transparency in the visible light region. However, due to the limited reserves of indium, the optoelectronic applications of ITO are severely restricted. Therefore, next-generation transparent electrodes consisting of the emerging 2D materials are highly desirable.

In this work, mica was used as the substrate, owing to its 2D layered structural nature with relatively weak interlayer interaction, which favors the 2D/2D van der Waals epitaxial (vdWE) growth. Furthermore, mica presents an atomically flat, chemically saturated, mechanically flexible, electrically insulating, and optically transparent surface, which is considered an ideal substrate for flexible transparent conductive electrodes [17,18]. Then, molecular beam epitaxial (MBE) growth of high-quality Bi_2_Se_3_ thin films has been realized on mica substrates, exhibiting a high (001) texture with an XRD rocking curve full-width at half-maximum (FWHM) of 0.088° and a mobility of 726 cm^2^/(Vs) at room temperature and up to 1469 cm^2^/(Vs) at 12 K. An optoelectronic device consisting of the Bi_2_Se_3_/mica structure as the 2D flexible TCE and PTB7:PC_71_BM as the light absorber was fabricated, producing a photocurrent of 2.75 mA/cm^2^ and an open-circuit voltage of 697 mV, demonstrating the feasibility of Bi_2_Se_3_ for optoelectronic applications.

## 2. Materials and Methods

Bi_2_Se_3_ was synthesized by the MBE technique on the mica 2D substrate. In addition, 5N purity Bi_2_Se_3_ pieces (purchased from American Elements, Los Angeles, CA, USA) were used as the evaporation source, loaded in a K-cell and heated up to 470 °C for the film deposition. The substrate temperature was maintained at ~340 °C, and the film growth rate was evaluated to be around 4.5 nm/min by ex-situ measurement of the film thickness with the help of an Alpha Step 500 profiler. The Bi_2_Se_3_/mica 2D material-based transparent conductive electrode was then covered by a thin layer of V_2_O_5_ as the hole-transporting layer by thermal evaporation, followed by spin-coating the PTB7:PC_71_BM absorber. Consequently, a calcium/aluminum electrode was evaporated onto the layers, forming an optoelectronic device.

The microstructure and crystal phase of the prepared Bi_2_Se_3_ thin films were characterized by high-resolution XRD and recorded on a Rigaku Smartlab 9 kW X-ray diffractometer (Rigaku Corporation, Tokyo, Japan), equipped with a Cu-Kα1 radiation source (λ = 1.5406 Å) and a two-crystal Ge (220) two-bounce hybrid monochromator (Rigaku Corporation, Tokyo, Japan). The film surface morphology was examined by AFM, which was performed on a Bruker NanoScope 8 (Billerica, MA, USA) in tapping mode using a silicon cantilever with a tip radius of less than 10 nm and resonance frequency of 341 kHz. Scanning electron microscopy (SEM) was also employed to investigate the morphology and the microstructure of the films, using a JEOL 6490 (JEOL Ltd., Tokyo, Japan) microscope by applying an accelerating voltage of 20 kV. Transmission electron microscopy (TEM) was employed to characterize the film microstructure, which was recorded through a JEM-2100F (field emission) scanning transmission electron microscope (JEOL Ltd., Tokyo, Japan) equipped with an Oxford INCA x-sight EDS Si(Li) detector (Oxford Instruments, Abingdon, UK)at an acceleration voltage of 200 kV. The film electrical properties were determined by Hall measurements using the four-probe van der Pauw method, performed on a Bio-Rad 5500 Hall system (Hercules, CA, USA) at room temperature using a permanent magnet with a magnetic field of 0.32 T, and conducted in a cryostat equipped with a CTI-Cryogenics 22 Refrigerator (Helix Technology Corporation, Mansfield, MA, USA) from room temperature to ~12 K under an electromagnetic field of 0.32 T. The work function of the Bi_2_Se_3_ thin film was deduced from ultraviolet photoelectron spectroscopy (UPS) measurements, which were conducted in ultra-high vacuum at 5 × 10^−8^ mbar, by irradiating with 21.22 eV photons (He I line). The photoelectric response of the prepared device was recorded on an Agilent B1500A semiconductor device parameter analyzer (Santa Clara, CA, USA). The photocurrent was collected under a simulated Air Mass 1.5 Global (AM 1.5 G) illumination, which was provided by a Newport Oriel Sol3A Solar Simulator (Irvine, CA, USA) with a 100 mW/cm^2^ radiance intensity.

## 3. Results and Discussion

**Figure 1:**
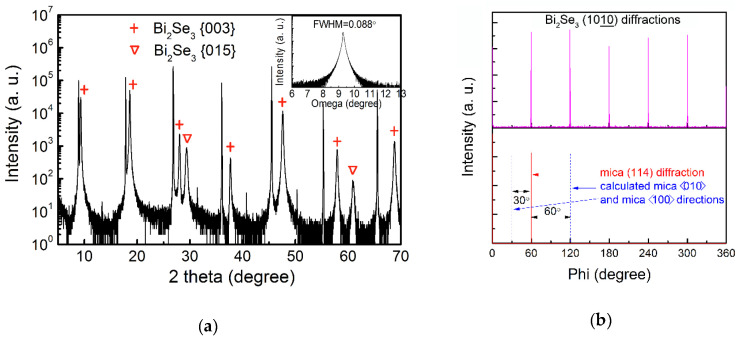
The XRD diffraction patterns of (**a**) 2θ-ω scan and (**b**) in-plane phi scan of Bi_2_Se_3_ on mica substrate. The inset in (**a**) gives the rocking curve.

Being a 2D layered material, high-quality Bi_2_Se_3_ can be deposited on various substrates, especially on 2D substrates, in spite of the large lattice mismatch. Figure 1 shows the XRD patterns of Bi_2_Se_3_ on the mica substrate. As indicated by the red crosses in Figure 1a, the predominant diffractions originated from the Bi_2_Se_3_ {003} family planes, suggesting that the Bi_2_Se_3_ film stacks vertically on the mica substrate along a highly c-axis-oriented direction. The presence of the Bi_2_Se_3_ {015} diffractions, as labeled by the inverted triangles, indicated that a certain quantity of crystals with the Bi_2_Se_3_ (015) planes parallel to the mica (001) surface simultaneously emerged. The inset in Figure 1a gives the XRD rocking curve, in which a full-width at half-maximum (FWHM) of 0.088° (~317 arc seconds) was attained, implying an excellent Bi_2_Se_3_ crystallinity and a perfect crystal alignment of vertical growth. Figure 1b shows the in-plane phi scans conducted on the Bi_2_Se_3_ (1010) plane and the mica (114) plane. Six sharp peaks separated by 60° showed up in the Bi_2_Se_3_ (1010) diffraction pattern, consistent with reports [19,20,21,22,23]. The observed six-fold symmetry, instead of three peaks expected for the trigonal system, was ascribed to the twin domains either stacking in the sequence -[ABCAB]- or -[CABCA]- [20]. Azimuthal diffractions of mica (114) and ($1\bar{1}4$) planes were superimposed on the same plot, from which the mica <100> and <010> directions on the mica (001) surface can be derived, as indicated by the blue dotted lines in Figure 1b [17]. The in-plane crystallographic relationship between Bi_2_Se_3_ and the mica substrate is then deduced as follows: Bi_2_Se_3_ (001) // mica (001) and Bi_2_Se_3_ <100> // mica <100>.

**Figure 2:**
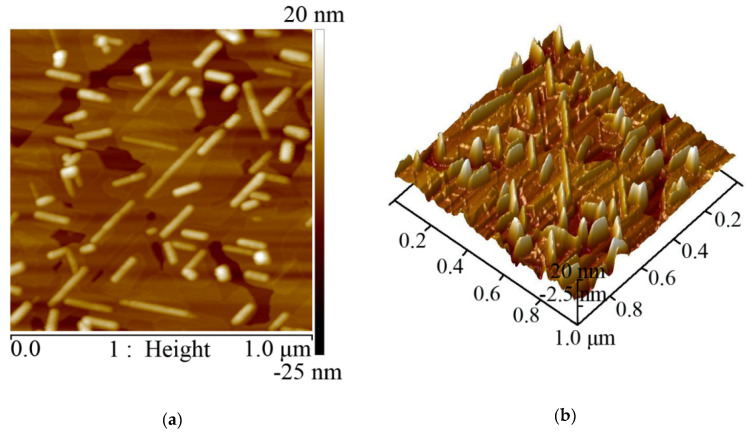
The AFM images of (**a**) top view and (**b**) perspective view of Bi_2_Se_3_ on mica substrate.

As shown in the AFM top view image in Figure 2a, rhombohedral-shaped planes were observed, suggesting the highly c-oriented van der Waals (vdW) epitaxial growth. At the same time, fence-like vertical planes emerged, as shown clearly in the AFM perspective view in Figure 2b. These fence-like planes are parallel to the edge of the rhombohedral planes and are oriented at 60° and 120° relative to each other.

**Figure 3:**
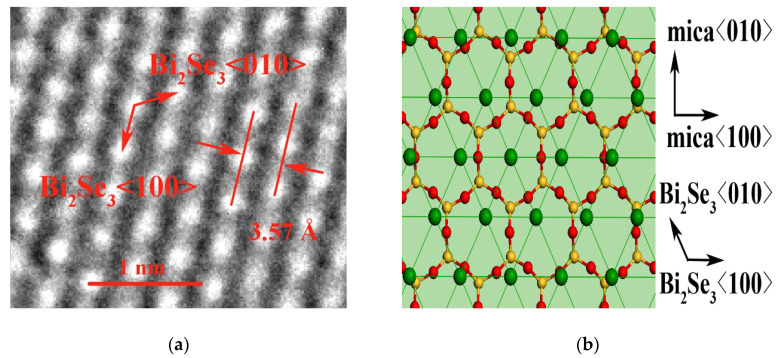

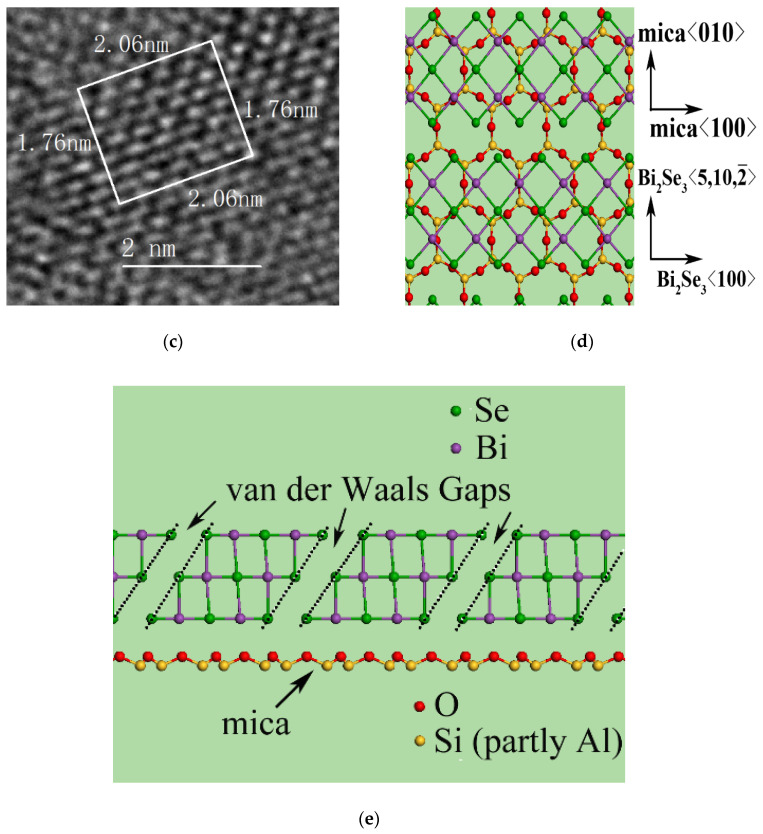
The TEM images and the interface models of Bi_2_Se_3_ on mica. (**a**,**b**) show the Bi_2_Se_3_ (001) // mica (001) planes; (**c**)–(**e**) give the Bi_2_Se_3_ (015) // mica (001) planes. In the interface models, O and Si (partly Al) atoms in mica are colored respectively by red and yellow, while Bi and Se atoms in Bi_2_Se_3_ are colored respectively by violet and green.

High-resolution TEM measurements were conducted to reveal the Bi_2_Se_3_ microstructure. The hexagonal atomic arrangement, as shown in Figure 3a, confirms the c-axis-oriented growth direction of the film. The average distance between the planes is found to be 3.57 Å, as indicated by the red arrows, signifying the lattice constant as ***a*** = 4.12 Å. Figure 3c shows the atomic arrangement within the vertical planes. The average spacing between the planes indicated by the white rectangle was found to be 2.20 Å, in good agreement with the calculated value of 2.21 Å between planes along the Bi_2_Se_3_ <$5,10,\bar{2}$> direction. The distance of 2.06 nm between the five atoms along the longer side of the white rectangle coincided nicely with the lattice along Bi_2_Se_3_ <500>, in which 5***a*** = 20.61 Å. These results confirm that the fence-like vertical planes are those that grew along the Bi_2_Se_3_ (015) normal. The crystallographic relationship of Bi_2_Se_3_ and the mica (001) surface at these different growth orientations is illustrated by using the interface models, as shown in Figure 3b,d,e.

For van der Waals epitaxial growth with a large lattice misfit between the epilayer and substrate, the matching condition can be evaluated via the coincidence lattice mismatch [17], δ, which is defined as (m*d*_epi_–n*d*_sub_)/n*d*_sub_, where *d*_epi_ and *d*_sub_ are the atomic distances of the epilayer and substrate, respectively. Lattice coincidence occurs when *d*_epi_/*d*_sub_ = n/m, where n and m are positive integers and n/m is the smallest non-reducible integral ratio [24,25]. Then, the concept of coincidence site lattice unit (CSLU) can be established as CSLU = *n*_x_ × *n*_y_, where *x* and *y* denote the directions along the substrate lattice vectors ***a*** and ***b***, respectively. It can be concluded that the smaller the CSLU, the better the lattice match between the epilayer and the substrate, as a small CSLU represents a larger number of coincidence sites per unit area [17]. For the case of planar growth, as shown in Figure 3b, the CSLU is 4 × 2 = 8. By contrast, for the vertical growth as illustrated in Figure 3d,e, the CSLUs are calculated to be 4 × 1 = 4 and 4 × 5 = 20 regardless of and counting the period of van der Waals gaps, respectively. It is apparent that the growth direction along the Bi_2_Se_3_ <$5,10,\bar{2}$> direction will develop a larger CSLU size when involving more van der Waals gaps. Therefore, the growth with a smaller CSLU of 4 × 1 will be favored, leading to the preferential growth directions along Bi_2_Se_3_ <100> and the Bi_2_Se_3_ (015) normal and producing the fence-like planes.

**Figure 4:**
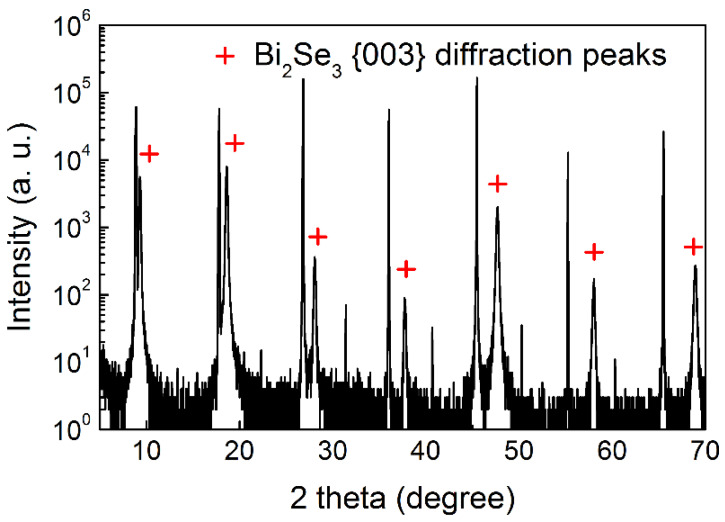
XRD diffraction pattern of Bi_2_Se_3_ on mica with buffer layer. The Bi_2_Se_3_ {003} family of planes are labeled by red crosses, while other peaks originate from the mica substrate.

**Figure 5:**
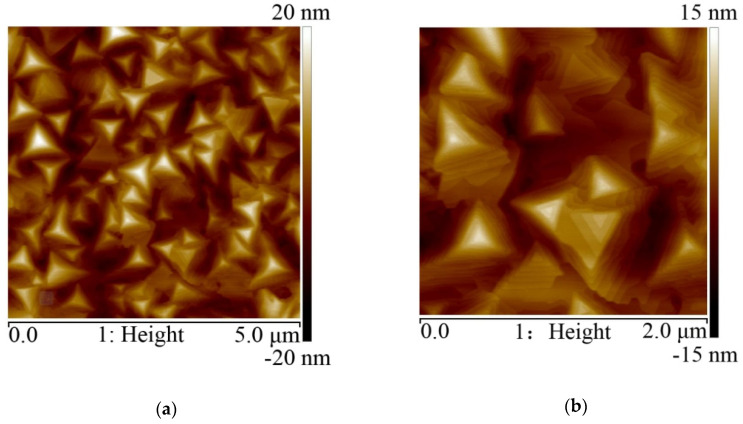
AFM images of Bi_2_Se_3_ on mica with an amorphous buffer layer. (**a**) is on a 5 × 5 μm^2^ scale; (**b**) is on a 2 × 2 μm^2^ scale.

The fence-like planes make the film surface uneven, e.g., a film 90 nm thick has a root-mean-square (rms) roughness of 17.8 nm on the scale of 5 × 5 μm^2^ (see Figure S1 in the Supplementary Materials). This would bring harm to the fabrication and performance of electronic devices. To eliminate these fence-like vertical planes, a thin amorphous Bi_2_Se_3_ buffer layer was prepared at room temperature, prior to the Bi_2_Se_3_ growth at high substrate temperature. Figure 4 shows the XRD diffraction patterns of Bi_2_Se_3_ with an amorphous buffer layer on the mica substrate. It was clearly observed that the Bi_2_Se_3_ {015} diffractions, corresponding to the fence-like vertical planes, totally disappeared. As displayed in the AFM images in Figure 5, the surface morphology of the prepared Bi_2_Se_3_ film of ~90 nm with a buffer layer of ~5 nm is greatly improved, reflected by the reduction in the rms roughness to 4.9 nm over an area of 5 × 5 μm^2^. Fence-like planes have totally disappeared, and triangular terraces are clearly observed, signifying that only the planar growth along Bi_2_Se_3_ <001> occurs. The buffer layer presenting as an amorphous state significantly weakens the interaction between the first stacking layer of Bi_2_Se_3_ and the mica surface, favoring the van der Waals epitaxial growth on top of it. However, it is also because of the much weaker interaction; the lateral growth alignment of van der Waals epitaxies will be weakened as well as reflected by the much broader peaks of the azimuthal phi scan with an average FWHM of 13.8 (see Figure S2 in the Supplementary Materials), which may contribute a large number of grain boundaries (GBs) in the film.

**Figure 6:**
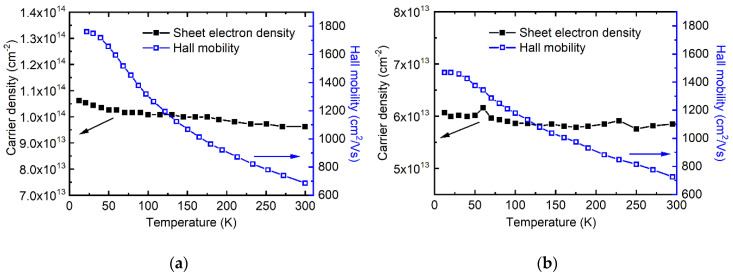
Sheet carrier density and mobility as a function of temperature for the Bi_2_Se_3_ film on mica substrate. (**a**) is without and (**b**) is with a buffer layer.

The transport performances of the carrier density and the mobility dependence on temperature for the Bi_2_Se_3_ thin film were investigated by employing low-temperature Hall measurement. Figure 6a,b show the electrical properties of 90 nm thick Bi_2_Se_3_ films on mica without and with a buffer layer, respectively. When grown directly on mica, the Bi_2_Se_3_ Hall mobility increased from 697 cm^2^/(Vs) at room temperature to 1761 cm^2^/(Vs) at ~12 K, while the sheet electron density remained nearly constant at about 1.0 × 10^14^ cm^−2^. By applying an amorphous Bi_2_Se_3_ buffer layer, the mobility at room temperature was slightly boosted to 726 cm^2^/(Vs) with a reduced sheet carrier density of ~5.9 × 10^13^ cm^−2^. The mobility increased at a near linear trend from 726 to 1469 cm^2^/(Vs) as the temperature fell. However, the increase in the mobility of Bi_2_Se_3_ with the buffer layer lagged that without the buffer layer when the temperature decreased below ~115 K. The fence-like planes in the Bi_2_Se_3_ film grown directly on mica could contribute to the higher carrier density via introducing a larger surface area. However, the lower the mobility in the low-temperature range of Bi_2_Se_3_ with the buffer layer can be ascribed to the poor lateral alignment of crystals as discussed above. The weak interaction between the amorphous buffer layer and the Bi_2_Se_3_ crystals on top, although leading to removal of the fence-like planes, also weakened the lateral growth alignment, which produced a number of GBs mirrored by the misoriented sub-micrometer-sized domains in the AFM images in Figure 5 and the broad FWHM of the XRD in-plane phi scan in Figure S2. According to [26,27], the GBs play an important role in the mobility decline of 2D MoS_2_ by means of interdomain scattering. In this study, the interdomain scattering arising from the misorientation of Bi_2_Se_3_ crystals on the buffer layer may contribute a lot to the slow rise in mobility with the decrease in temperature in the low-temperature range. By contrast, the lattice scattering may dominate the mobility variation in the Bi_2_Se_3_ crystals directly grown on mica, as they are perfectly aligned and connected laterally. The electrical transport property of 2D materials requires further investigation.

**Figure 7:**
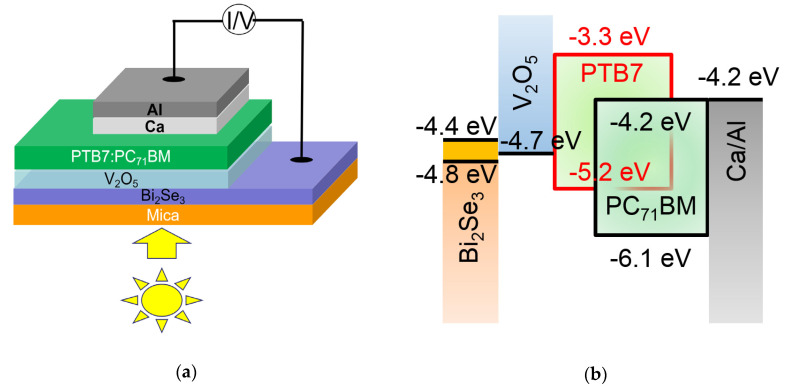

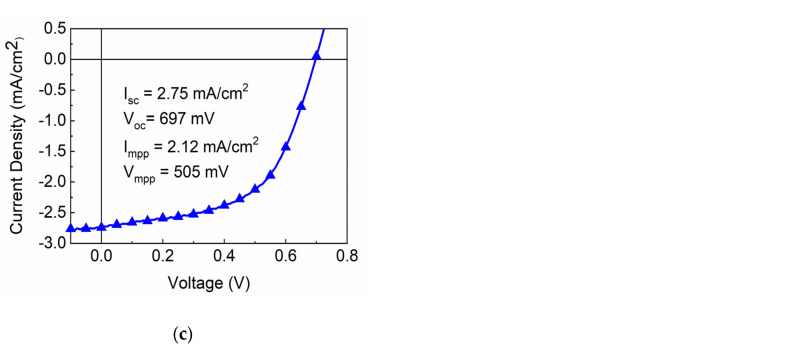
The Bi_2_Se_3_-based organic optoelectronic device. (**a**) Schematic diagram of the device structure; (**b**) energy band alignment; (**c**) *I*–*V* characteristics.

An optoelectronic device comprising the Bi_2_Se_3_/mica structure and organic absorber was fabricated, as shown in Figure 7a. The energy band alignment between the device components is illustrated in Figure 7b, in which the work function of the Bi_2_Se_3_ thin film is measured as 4.43 eV by using UPS (see Figure S3 in the Supplementary Materials). Figure 7c displays the *I*–*V* characteristics of the device, in which a short-circuit photocurrent of 2.75 mA/cm^2^ and an open-circuit voltage of 697 mV are achieved. It is noteworthy that the values of photoelectric parameters are relatively lower compared to the conventional PTB7:PC_71_BM-based solar cells, for which TCEs such as ITO coated on glasses are commonly used as the substrates instead of Bi_2_Se_3_ deposited on the mica. The absorption range of Bi_2_Se_3_ covers the broad visible light region due to the small bandgap of Bi_2_Se_3_, causing a reduced amount of light reaching the active layer (PTB7:PC_71_BM) of the device to generate photocarriers. To address this issue, the thickness of Bi_2_Se_3_ should be further optimized or the opaque aluminum electrode should be replaced with transparent electrodes so that the light can be transmitted from both sides. The interface quality of the devices should also be investigated as Bi_2_Se_3_ coated on flexible mica will exhibit a different strain on the functional layers in the optoelectronic devices compared to the devices using the conventional rigid ITO-coated glass substrates. The presence of interfacial defects leads to carrier recombination, resulting in a poor fill factor of devices. Some interfacial passivation strategies [28,29] can be performed to relieve their negative impact on the device performance. Nevertheless, the demonstration of using Bi_2_Se_3_ in PTB7:PC_71_BM-based optoelectronic devices in this work aims to pave the way to explore 2D transition metal dichalcogenides and exhibit their potential in the optoelectronic applications.

## 4. Conclusions

High-quality Bi_2_Se_3_ thin films have been prepared on a mica substrate via vdW epitaxial growth. The film exhibited a highly c-axis-oriented growth with an extraordinary crystallinity, reflected by a narrow XRD rocking curve FWHM of ~317 arc seconds. At the same time, fence-like planes with Bi_2_Se_3_ (015) parallel to the mica (001) surface emerged, which was explained by using the interface model associated with the CSLU concept developed especially for vdWE. To eliminate the fence-like planes, an amorphous Bi_2_Se_3_ buffer layer prepared at the substrate temperature of room temperature was applied, prior to the Bi_2_Se_3_ crystal growth. As a result, only the Bi_2_Se_3_ (001) growth direction with vdW gaps parallel to the substrate showed up, with a smoother surface than that without the amorphous buffer layer. However, this suppression in the fence-like planes sacrifices to a certain degree the crystal lateral growth alignment, mirrored by a broadening phi scan peak FWHM. A high Hall mobility of 1761 cm^2^/(Vs) for Bi_2_Se_3_ on mica was obtained at ~12 K, and 697 cm^2^/(Vs) at room temperature with a nearly constant sheet electron density of ~1.0 × 10^14^ cm^−2^. By applying an amorphous Bi_2_Se_3_ buffer layer, the carrier density was reduced to about half that without the buffer layer. The mobility at room temperature was boosted to 726 cm^2^/(Vs), and linearly increased to 1469 cm^2^/(Vs) as the temperature dropped to 12 K. An optoelectronic device consisting of a Bi_2_Se_3_/mica TCE, organic absorber, and Ca/Al metal back electrode was designed and prepared, generating a short-circuit photocurrent of 2.75 mA/cm^2^ and an open-circuit voltage of 697 mV under one sun irradiation. The results demonstrate the great potential of 2D Bi_2_Se_3_ along with mica for flexible optoelectronic applications.

## Supplementary Materials

The following are available online at https://www.mdpi.com/2079-4991/10/9/1653/s1, Figure S1: AFM images of 90 nm-thick Bi_2_Se_3_ film with an rms roughness of 17.8 nm, Figure S2: In-plane phi scan of Bi_2_Se_3_ on mica with buffer layer, Figure S3: UPS spectrum of Bi_2_Se_3_.
