# Supplementary material for: 2D Bi2Se3 van der Waals Epitaxy on Mica for Optoelectronics Applications
Source: Nanomaterials (Basel). 2020 Aug 22;10(9):1653. doi: 10.3390/nano10091653 (PMC7558585; doi:10.3390/nano10091653)
Supplement: Supplementary file 1 [file nanomaterials-10-01653-s001.pdf]

2D Bi_2Se_3 van der Waals Epitaxy on Mica for Optoelectronics Applications

Shifeng Wang [1,2,*], Yong Li [1,2], Annie Ng [3,*], Qing Hu [4,5], Qianyu Zhou [1], Xin Li [1] and Hao Liu [1]

[1] Department of Physics, Innovation Laboratory of Materials for Energy and Environment Technologies, College of Science, Tibet University, Lhasa 850000, China; xzuliyong@utibet.edu.cn (Y.L.); zhouqianyu@utibet.edu.cn (Q.Z.); lixin@utibet.edu.cn (X.L.); liuhao@utibet.edu.cn (H.L.)
[2] Institute of Oxygen Supply, Center of Tibetan Studies (Everest Research Institute), Tibet University, Lhasa 850000, China
[3] Department of Electrical and Computer Engineering, Nazarbayev University, Nur-Sultan 010000, Kazakhstan
[4] School of Environmental Science and Engineering, Southern University of Science and Technology, Shenzhen 518055, China; huq@sustech.edu.cn
[5] Engineering Innovation Center of Southern University of Science and Technology, Beijing 100083, China
* Correspondence: wsf@utibet.edu.cn (S.W.); annie.ng@nu.edu.kz (A.N.)

Supplementary Materials

S1. AFM images of Bi_2Se_3 film of 90 nm thick.

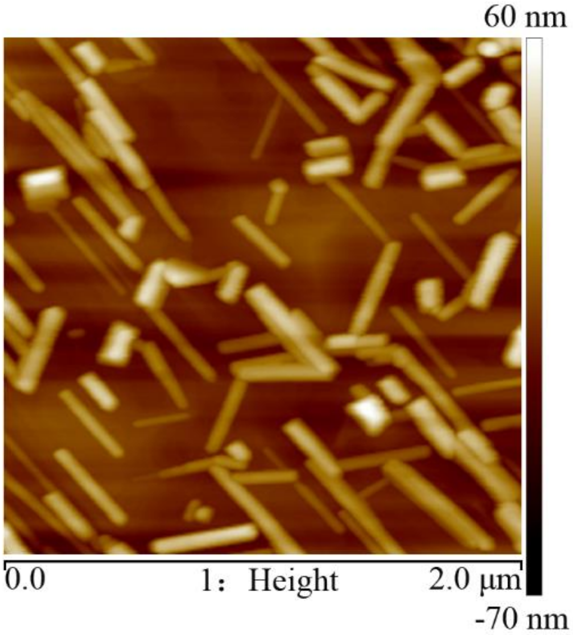

Figure S1. AFM images of Bi_2Se_3 film of 90 nm thick with a rms roughness of 17.8 nm.

S2. In-plane phi scan of Bi_2Se_3 on mica with buffer layer.

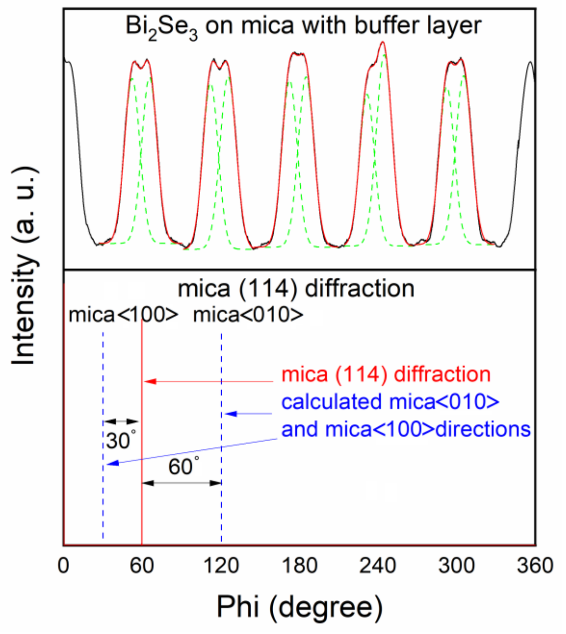

Figure S2. In-plane phi scan of Bi_2Se_3 on mica with buffer layer. Each diffraction peak can be deconvoluted into two peaks with an average FWHM of 13.8° indicated by the green dashed lines.

S3. UPS measurement of Bi_2Se_3.

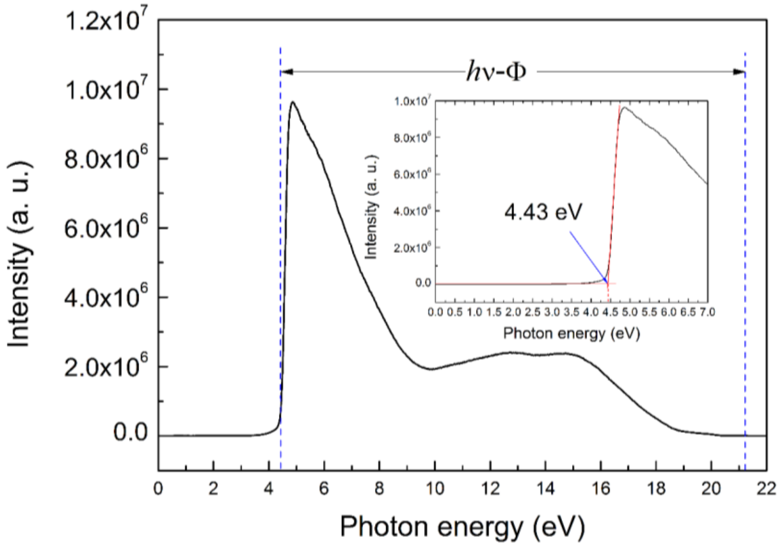

Figure S3. UPS spectrum of Bi_2Se_3. The work function is calculated to be 4.43 eV.
